# The Comparison of Classical Statistical and Machine Learning Methods in Prediction of Thrombosis in Patients with Acute Myeloid Leukemia

**DOI:** 10.3390/bioengineering12010063

**Published:** 2025-01-13

**Authors:** Ilija Doknić, Mirjana Mitrović, Zoran Bukumirić, Marijana Virijević, Nikola Pantić, Nikica Sabljić, Darko Antić, Živko Bojović

**Affiliations:** 1 Faculty of Sciences, University of Novi Sad, 21000 Novi Sad, Serbia; 2 Clinic of Hematology, University Clinical Center of Serbia, 11000 Belgrade, Serbiamarijana.virijevic@yahoo.com (M.V.); nsabljic19@gmail.com (N.S.); darko.antic1510976@gmail.com (D.A.); 3 Faculty of Medicine, University of Belgrade, 11000 Belgrade, Serbia; 4 Faculty of Informatics and Computing, Singidunum University, 11000 Belgrade, Serbia; zbojovic@singidunum.ac.rs

**Keywords:** thrombosis, acute myeloid leukemia, machine learning, neural networks, classical statistical methods

## Abstract

Thrombosis is one of the most frequent complications of cancer, with a potential impact on morbidity and mortality, particularly those with acute myeloid leukemia (AML). Therefore, effective thrombosis prevention is a crucial aspect of cancer management. However, preventive measures against thrombosis may carry inherent risks and complications. Consequently, the application of thrombosis prevention should be limited to patients with a reasonable risk of developing thrombosis. This thesis explores the potential of data science (DS) methods for predicting venous thrombosis in patients with acute myeloid leukemia. In order to ascertain which patients are at risk, statistical and machine-learning (ML) algorithms were employed to predict which patients with leukemia will develop thrombosis. Multilayer Perceptron (MLP) was found to be the best fit among the models evaluated, achieving the C statistic of 0.749. We examined which attributes are significant and what role they play in prediction and found six significant parameters: sex of the patient, prior history of thrombotic event, type of therapy, international normalized ratio (INR), Eastern Cooperative Oncology Group (ECOG) performance status, and Hematopoietic Cell Transplantation-specific Comorbidity. These findings suggest that subtle DS techniques can improve the prediction of Thrombosis in AML patients, thereby aiding in individual treatment planning.

## 1. Introduction

Today, thrombosis represents one of the leading causes of morbidity and mortality in cancer patients. In the case of acute leukemias, thrombosis risk ranges from 2% to 22% [1,2,3,4,5] and may exceed that of solid tumors. Factors like leukemia type, Central Venous Line (CVL) use, and certain types of therapy contribute to this variability. Although primary thromboprophylaxis may be beneficial, its use is limited by thrombocytopenia, bleeding risks, and a lack of guidelines [6]. Research shows mixed results on thrombosis risk factors, with predictors including sex, age, CVL, past thrombosis, platelet counts, D-dimer levels, Disseminated Intravascular Coagulation (DIC), and specific genetic mutations. A suitable risk assessment model for acute myeloid leukemia patients is needed. Today, we see the increasing usage of machine learning and artificial intelligence (AI) to develop a risk assessment model based on data analytics for thrombosis, as well as in other fields of medicine.

AI techniques like deep learning and decision trees enhance medical diagnostics by extracting key variables from big data. AI shows promise in thrombotic diseases, including hereditary thrombophilia [7]. Evaluating ML algorithms, especially for time series, demonstrates the effectiveness of supervised and unsupervised methods across various dataset sizes [5]. Despite the advances in ML and AI, it is important to determine the best approach based on data complexity, accuracy, interpretability, and available resources for each case study [8]. Therefore, it is necessary to compare results obtained by ML with classical statistical methods; balancing prediction accuracy with interpretability is crucial for achieving reliable forecasts and optimal decision-making. Conducting a comparative analysis enables the determination of which model best fits the data, supporting informed decisions in healthcare.

A comparative analysis of classical statistical models (e.g., Autoregressive Integrated Moving Average—ARIMA) and machine learning models (e.g., Long-Short-Term Memory—LSTM, Convolutional Neural Network—CNN) was conducted [4] for TB/HIV coinfection prediction, showing that deep learning models like Bidirectional LSTM and CNN-LSTM outperformed classical methods. A similar study [3] on lung cancer survival prediction with 10,000 patients found that deep neural networks achieved the highest accuracy (88.58%), aiding healthcare providers in cost and treatment management. This comparison aims to help researchers identify the most effective models for their predictive needs. No single model fits all situations, so choosing the best one for each use case is crucial. Machine learning (ML) offers flexibility and scalability, making it suitable for tasks such as diagnosis and survival prediction. On the other hand, classical statistical methods are often more effective when the number of cases exceeds the number of variables. Integrating both ML and traditional statistical methods may provide better outcomes for a more comprehensive approach to healthcare solutions [9]. A combined approach integrating both methods is recommended for optimal healthcare solutions.

Our research builds upon the article “Venous Thromboembolism in Patients with Acute Myeloid Leukemia: Development of a Predictive Model” [10]. This retrospective cohort study analyzed adult patients with newly diagnosed AML, using univariate and multivariable logistic regression to estimate binary outcomes and identify potential predictors. The analysis identified five predictors: patient sex, prior history of thrombotic events, international normalized ratio (INR), Eastern Cooperative Oncology Group performance status (ECOG), and intensive therapy. The predictive model demonstrated an area under the curve (AUC) statistic of 0.68, which provides valuable insights into potential approaches to thromboprophylaxis in patients with AML.

However, we must note that parameters on vastly different scales were not subjected to preprocessing to ensure they were within the same range. Further, the data was not divided into a training set and a test set; rather, the model was trained on the entire dataset, and the results were reported on the whole dataset. It may be fine when working with logistic regression due to its linear nature. However, when using more complex non-linear models, it is advisable to use a test set as a guard against overfitting. Finally, an extensive parameter search was not performed, meaning that regularization and related parameters were not tried.

## 2. Materials and Methods

### 2.1. Patient Selection and Data Collection

The data were gathered at the Clinic for Hematology at the University Clinical Center of Serbia between 2009 and 2021. All adults (≥18 years of age) with a newly diagnosed AML who were diagnosed and treated in the Clinic for Hematology at the University Clinical Center of Serbia were included. The retrieval of information and publication of these results were approved by the Institutional Review Board of the University Clinical Center of Serbia (protocol number III 41,004).

The diagnosis of AML was confirmed through cytological, flow cytometry, and cytogenetic analyses, following the criteria established by the World Health Organization and European Leukemia Net [11,12]. Patients with acute promyelocytic leukemia were excluded from this study. Participants were monitored from the point of diagnosis until the occurrence of VTE, death, or up to 6 months post-diagnosis. Patients were treated in an intensive (“3 + 7” induction followed by intermediate-dose cytarabine [IDAC] consolidation and allogeneic hematopoietic stem cell transplantation), non-intensive (azacytidine, low-dose chemotherapy), or supportive manner [1,11].

Data collection encompassed demographic information (age, sex), body mass index (BMI), smoking status, comorbid conditions (including prior thrombosis), concurrent treatments, ECOG performance status (PS), Hematopoietic Cell Transplantation-specific Comorbidity Index (HCT_CI), baseline laboratory values (complete blood count, fibrinogen, prothrombin time [PT], International Normalised Ratio [INR], activated partial thromboplastin time [APTT], D-dimer, lactate dehydrogenase [LDH]), leukemia-specific parameters (cytogenetics, molecular genetics [FLT3, NPM1], flow cytometry), the type (intensive, non-intensive, or palliative) and phase of leukemia treatment, presence of a central venous line (CVL), Khorana and Al-Ani scores, and concurrent COVID-19 status. All laboratory values, along with comorbid conditions, concurrent treatments, and smoking status, were recorded on the day of diagnosis or within a 3-day window prior.

### 2.2. Surgical/Medical Procedure

The primary outcome was the development of symptomatic, imaging-confirmed venous thromboembolism (VTE), which included deep venous thrombosis (DVT) of the upper and lower limbs, pulmonary embolism (PE), thrombosis at unusual sites (such as cerebral and portal vein thrombosis), and symptomatic CVL-related thrombosis (DVT at any location associated with a CVL) [13]. DVT diagnosis was based on compression ultrasound evidence of a thrombus. Acute PE was defined by the presence of filling defects on computed tomography pulmonary angiography.

### 2.3. Machine Learning-Based Research Methodology

Prior to undertaking any processing steps, it is essential to divide the data into training and test sets, as the latter must remain intact until the model has been validated. Otherwise, information about it would be leaked, i.e., the values in the test set could be inferred from the train set, thus leading to biased validation. The data was divided into two sets under a 70:30 distribution, meaning 70% of patient data (*n* = 438) was used for fitting the model, and 30% (*n* = 188) was used for validation. Splits were stratified so that there were equal proportions of positive cases in both the training and testing datasets.

Afterward, all features were scaled to a uniform range with precision using scaling specifications determined on the training dataset and subsequently applied to both the training and test sets. Extensive data visualization and interpretation were conducted to determine the normality of data, i.e., to discover potential outliers or irregularities. Features with more than 5% missing values were excluded from the analysis, and the remaining ones were imputed: continuous variables were imputed with median, in contrast to categorical variables that were imputed by most frequent values. Highly correlated features and those with a Variance Inflation Factor (VIF) greater than five were excluded to mitigate multicollinearity.

**Figure 1:**
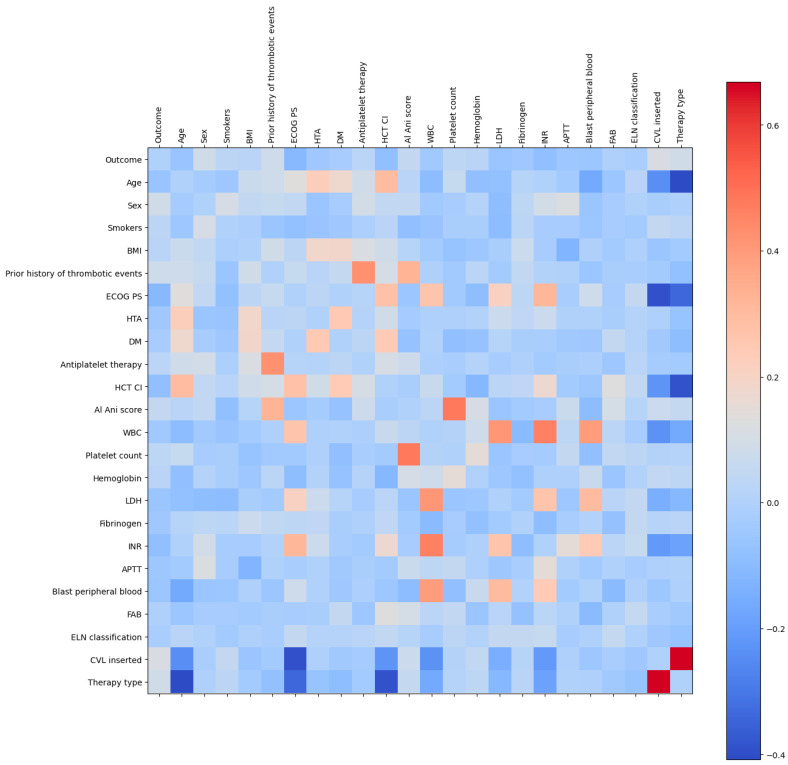
Colored correlation matrix. The strength of the correlation is represented by the corresponding color.

The subsequent image (Figure 1) illustrates the correlation between the remaining columns. It can be observed that there is a strong positive correlation between the type of therapy and CVL; therefore, CVL was excluded.

The final stage of the preprocessing phase was the selection of features. This was achieved through the application of univariate logistic regression to each feature, with the objective of calculating their respective *p*-values. The *p*-value indicates the probability of obtaining an extreme or more extreme result than the observed result when a model that embodies the null hypothesis is employed.

**Figure 2:**
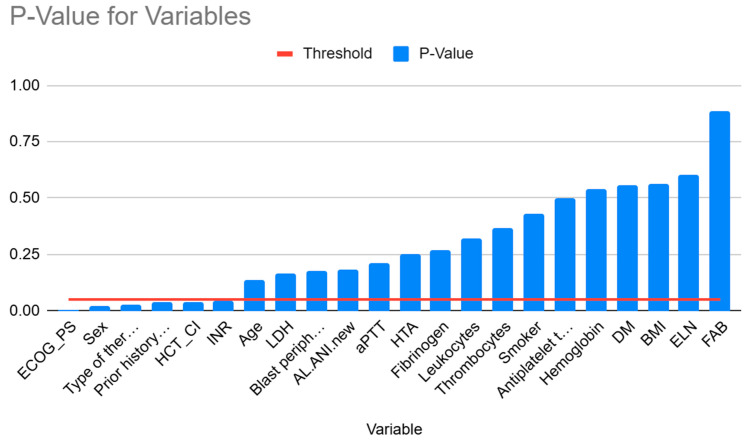
The *p*-value for different variables. The threshold is the common alpha value of 0.95.

The results of the univariate logistic regression, applied to all parameters, are presented in the Figure 2:

It can be observed that only six parameters have a *p*-value that is less than 0.05, which is the standard threshold. The parameters mentioned above are ECOG_PS, the patient’s sex, the type of therapy, previous thrombosis, HCT_CI, and INR. Furthermore, forward and backward selection was conducted, which also demonstrated that utilizing more than six attributes has a detrimental impact on performance. To illustrate, we employed Naive Bayes with a forward selection process and these six parameters as the basis. The process augmented the model with three additional parameters: ‘LDH,’ ‘Blast peripheral blood,’ and ‘aPTT.’ However, the model with the augmented parameters exhibited inferior performance compared to the original model, with an AUC score of 72.24% compared to 73.49%. A similar pattern was observed with Logistic Regression and K-Nearest Neighbors.

A further advantage of a reduced number of parameters is that the model is more comprehensible and simpler to use. Greater explicitness facilitates a more accurate understanding of the parameters responsible for positive diagnoses or positive predictions in general. Additionally, the reduction in parameters facilitates the development of a service that is more readily applicable in practice, as medical professionals would be required to collect less data.

## 3. Results

### 3.1. Descriptive Statistics

We included 626 patients diagnosed with AML, 348 males (55.6%) and 278 females (44.6%), with a mean age of 55.1 ± 13.4 years. Thrombosis was found in 72 patients (11.5%), 49 of whom were males (68.1%). The detailed descriptive statistics are shown in Table 1 of Mitrovic et al., summarizing the demographic and clinicopathological features of the patient cohort [10].

### 3.2. Comparative Performance Analysis of Different Metrics for the ML Classifier

The following algorithms were employed in the training process:

- Logistic regression
- Ridge regression
- Naive Bayes
- Support Vector Machines (SVM)
- Random forest (RF)
- K-Nearest Neighbors (KNN)
- Neural networks i.e., Multilayer Perceptron (MLP)

The rationale behind the selection of these models is twofold. Firstly, they are among the most practical and widely utilized models in the field, and it was hypothesized that they would be effectual for thrombosis prediction. Secondly, medical data typically have a low number of instances for machine learning standards, a characteristic that favors the deployment of algorithms such as KNN, SVM, and Naïve Bayes [14]. Random Forest was selected as a more reliable variant of Decision Trees. While Decision Trees can identify interesting decision rules, they remain susceptible to overfitting. Ridge regression is a more flexible and powerful version of Logistic Regression, which allows for L2 regularization. The selection of neural networks, known for their flexibility and adaptability to varying dataset sizes, was guided by these qualities.

The SVM was unable to fit the data and exhibited severe underfitting, while the Random Forest and K-Nearest Neighbors demonstrated overfitting to an equivalent degree. Consequently, we ceased utilizing these models. The fact that the RF algorithm is able to fit the entire training data further perfectly illustrates the importance of splitting the data into two categories.

Hyperparameter tuning, which is one of the most comprehensive parts of model training, was conducted using a grid and random search. Grid search trains a model with each combination of hyperparameter values and returns the model with the highest optimization metric. It is a straightforward and widely used optimization approach. However, it has been empirically proved that random search is more efficient since, for most data sets, only a few of the hyper-parameters really matter, but different hyper-parameters are important in different data sets. This phenomenon makes grid search a poor choice for configuring algorithms for new data sets [15]. Nevertheless, we tried and compared both approaches.

Given the class imbalance present in our dataset, we conducted experiments to evaluate the effectiveness of various training techniques for handling unbalanced classes. Cost-sensitive learning, which assigns higher weights to minority classes, penalizes the algorithm more heavily for errors involving these classes. Another commonly used approach is upsampling the minority classes. However, simply duplicating the same data instances is often less effective. To address this, we employed the Synthetic Minority Oversampling Technique (SMOTE) [16], which generates synthetic minority class instances by interpolating within the feature space occupied by the minority classes. Despite these efforts, neither method resulted in improvements in the Area Under the Curve (AUC), which was the primary metric used for optimization in this study.

An essential method for addressing this task was adjusting the threshold for prediction. By default, this threshold is set to 0.5, classifying instances with prediction values above 0.5 as positive and those below 0.5 as negative. However, this default setting often requires adjustment, particularly in the context of imbalanced datasets. In our study, we determined that the threshold needed to be significantly lowered to achieve a balance between positive and negative predictions. Without this adjustment, all patients would have been classified as “not-at-risk,” reflecting the dominance of the majority class.

The reported results are based on the evaluation of the model on previously unseen test data. The following table presents the performance metrics for the algorithms that have successfully passed the first stage of training. The prediction threshold is the same for all algorithms and equals 0.15.

In the table above, Logistic Regression refers to the implementation employed by [9] The researchers utilized SPSS Statistics 30.0, which employs the Newton-Raphson method without regularization. This algorithm, in contrast to SVM, RF, and KNN, achieved a decent performance but was outweighed by all the other models. This result does not necessarily indicate that logistic regression is inherently less efficient; rather, it reflects the limitations of this particular implementation, which lacks the flexibility to adapt to specific problem requirements. Ridge Regression exemplifies this point, as it only differs from standard logistic regression by incorporating L2 regularization. The addition of L2 regularization introduces hyperparameters, enabling greater flexibility and improved performance in various contexts.

Naive Bayes also outperformed Logistic Regression, further demonstrating its effectiveness on smaller datasets [17]. The highest AUC score was achieved by the neural network. While this result highlights the potential superiority of modern machine learning models over classical statistical methods, the interpretation is not straightforward. First, the neural network used in this study was relatively small, consisting of only two layers with 12 and 14 neurons, respectively. Second, the MLP exhibited instability and sensitivity to initialization. Training the model with different random seeds often resulted in significant variations in performance. Notably, for a considerable portion of the training process, the MLP achieved lower AUC scores than Ridge Regression and Naive Bayes. While noteworthy, the eventual superior performance of the MLP was comparable to that of classical statistical methods.

**Figure 3:**
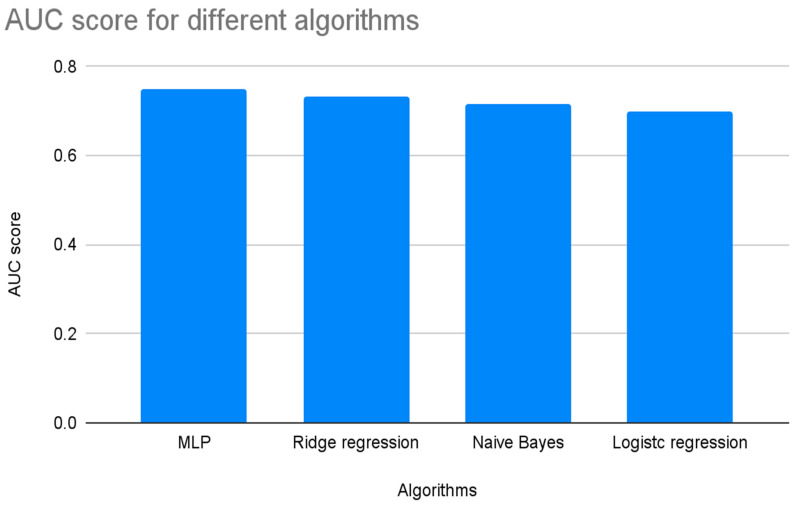
The bar graph displays AUC scores for algorithms deployed. MLP shows the highest AUC score at 0.749, followed by Ridge Regression (0.734), Naive Bayes (0.716), Logistic Regression (0.699), and K-Nearest Neighbors (0.581).

A 5 × 2 cross-validation test was conducted to assess whether the difference between classical statistical methods and machine learning approaches is statistically significant. This test is particularly recommended for scenarios where learning algorithms are computationally efficient enough to be executed multiple times. The results revealed that the performance gap between the best-performing neural network and the least effective logistic regression model was not statistically significant. From a theoretical perspective, this implies that no substantial improvement can be claimed. However, from a practical data science standpoint, the nearly 5-percentage-point difference in performance highlights the clear superiority of neural networks. Figure 3 illustrates these differences using a bar graph.

The AUC score was selected as the primary optimization metric because it reliably measures the underlying predictive power of a model and remains unaffected by variations in the prediction threshold [18]. In contrast, other metrics, such as sensitivity and specificity, are highly variable and heavily dependent on the chosen threshold. This does not diminish their importance; on the contrary, they provide valuable insights. However, it is crucial to contextualize these intrinsic metrics by comparing them with extrinsic metrics. Intrinsic metrics assess intermediary objectives and are relatively easy to compute, whereas extrinsic metrics evaluate the model’s impact on the final objective, which is significantly more challenging to measure [19]. In this study, intrinsic metrics are presented in Table 1, while an example of an extrinsic metric would involve assessing how the model improves patient health outcomes. Proper evaluation of such extrinsic metrics would require deploying the model in real-world settings and conducting a thorough analysis of its impact on clinical decision-making and patient care.

In our case, as in most medical scenarios, sensitivity is prioritized over specificity. This preference reflects the greater importance of minimizing false negatives: it is preferable to identify a healthy patient as sick and subject them to additional evaluations rather than misdiagnosing a sick patient as healthy and consequently failing to provide the necessary care. However, determining the optimal prediction threshold is critical to achieving a balance. This balance must be struck not only between sensitivity and specificity but also between treated and untreated patients, ensuring both accurate predictions and meaningful clinical outcomes.

**Figure 4:**
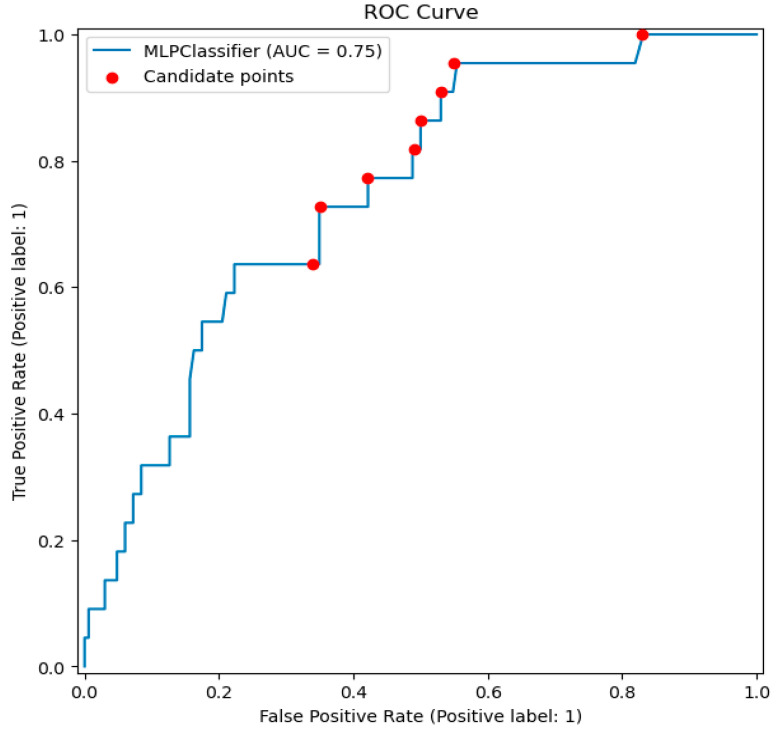
The ROC curve for the best-fitted model and candidate points identified as Pareto-dominant within the interval where the true positive rate is prioritized.

Although identifying the optimal prediction threshold is an iterative process that depends heavily on the underlying algorithm and requires extensive medical expertise, selecting appropriate candidates is relatively straightforward. This process involves plotting the Receiver Operating Characteristic (ROC) curve and identifying points along the curve known as Pareto-dominant points. These points are characterized by having the same specificity as all other points on the same horizontal line but achieving higher sensitivity, making them objectively superior. The ROC curve and corresponding Pareto-dominant points for the MLP algorithm are shown in the Figure 4.

### 3.3. Model Explainability

The performance of the model is one of many considerations when developing machine learning models. Nevertheless, it is also important for models to be able to explain the rationale behind specific decisions. Such insight enables researchers to understand the root cause of the problem more profoundly, thereby facilitating the implementation of effective solutions. In the event that a model indicates that a patient is at elevated risk of developing a disease, it would be highly beneficial if the model were to explain, thereby giving the affected patients the right to know the rationale behind specific decisions.

A straightforward approach to determining the contribution of each parameter is to generate a partial dependence plot. These plots are constructed by taking a single row instance and repeatedly modifying one of its features while maintaining all others constant. Predictions are then made with the modified instance and stored. Finally, the predictions are plotted against different values of the modified parameter, resulting in a plot that illustrates how the prediction value changes as the parameter value changes. This approach, however, carries a significant risk of yielding instances that do not align with the broader data distribution patterns. To illustrate, if one were to alter the age of a patient while maintaining other variables constant, the resulting data would be implausible in a real-world context.

**Figure 5:**
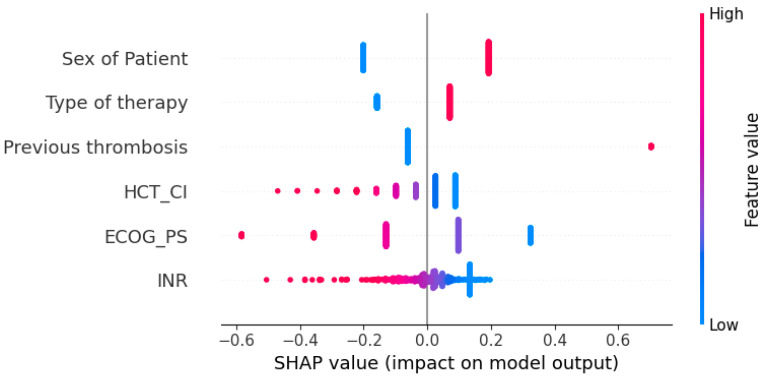
The summary plot of SHAP values. The horizontal axis represents the impact on the model output, while the color represents the height of the feature values. The length of the line symbolizes the number of instances within a certain group. So, the upper left line for the sex of the patient is colored blue, meaning it has a lower value. In our binary case, this line represents women. It is on the left side of the central line, indicating that women have a negative tendency to develop thrombosis. Finally, the length of the line suggests that there are slightly fewer women than men in the sample.

**Figure 6:**
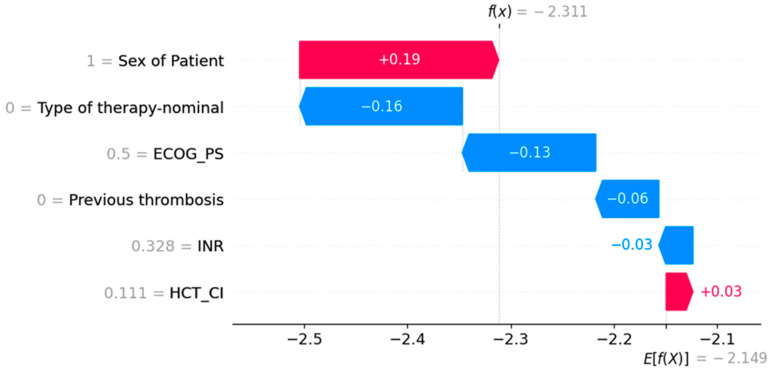
The waterfall plot of SHAP values. The plot explains a prediction of an individual patient. In this particular case, the model output is −2.311, which is slightly lower than the average output of −2.149. The patient’s HCT_CI slightly increases the chances of developing thrombosis, while being a male increases it by a big margin. However, when it comes to other factors, the patient has desirable values, which pushes prediction to the lower spectrum.

An approach offering a slight remedy to this problem is SHAP values (Figure 5 and Figure 6). The concept of Shapley values is inspired by game theory, and their primary purpose is to determine the contribution of each factor in a model in order to identify those with the greatest impact. These values are calculated iteratively, taking into account all possible combinations of the factors of interest, as well as their order. To exemplify, if the objective is to evaluate the significance of the following variables: (1) age, (2) gender, and (3) tumor size, the ML model’s prediction will be analyzed in relation to the impact of each variable’s inclusion or exclusion, in conjunction with the other two variables. This process will be repeated until all potential combinations of variable presence/absence and their order have been considered. At the end of each combination, we obtain the Shapley value for a given variable, and the average of these values, calculated from all possible combinations, represents the final Shapley value for the variable in question [20].

The analysis of feature influence shows the following:

- Men have a higher risk of developing thrombosis.
- Lower ECOG_PS scores are associated with a greater likelihood of developing thrombosis.
- Previous thrombosis, although rare, is the strongest predictor of developing the next thrombosis.
- Intensive therapy, which was often applied, is correlated with a higher probability of venous thrombosis.
- INR and HCT_CI are lower in patients who are at higher risk of venous thrombosis.

## 4. Discussion

In our study, the neural network emerged as the best-performing model. This superior performance can be attributed, in part, to the inherent flexibility of neural networks during hyperparameter tuning. A key aspect of this flexibility is the ability to adjust the network’s size significantly, enabling it to adapt effectively to the dataset’s characteristics. In this case, we designed a relatively small neural network, which was well-suited to the dataset. It is important to note that the dataset size is considered small by machine learning standards, rather than by medical research standards.

Ridge regression follows the neural network as the second-best model. In contrast to logistic regression, it utilizes an L2 penalty that improves the model’s performance by preventing overfitting, making it more robust in handling the dataset. Following Ridge Regression, the plain Naive Bayes algorithm ranked third in terms of performance, outperforming the Logistic Regression. This result is not surprising given the nature of the dataset, which is relatively small and contains only a few informative features. In such cases, Naive Bayes can often excel due to its simplicity and efficiency, as it does not require large amounts of data to perform well.

The present study demonstrates methods for enhancing the efficacy of contemporary medical applications, thereby facilitating more dependable and precise estimations. Many of the alterations introduced by these techniques are subtle, and their use does not necessitate extensive technical expertise on the part of healthcare practitioners. For instance, automated algorithms can process large datasets, thus enabling even those with limited technical skills to derive meaningful insights. Despite the seeming simplicity of these modifications, they can lead to substantial improvements in the accuracy and reliability of medical predictions, as well as in the efficiency with which healthcare services are delivered.

Furthermore, established industry standards govern the presentation and interpretation of findings derived from data-driven methods. Specifically, it is recommended that the data be divided into a training set and a test set in a standardized way. The division of the data should be made in a way that ensures the training set and test set are as similar as possible in terms of the percentage of each class. Providing these exact data splits to other clinicians and researchers ensures that results are not only scientifically sound but also transparent and interpretable.

Our current research highlights the importance of considering multi-parameter metrics that evaluate a model or system based on multiple parameters or factors. More than single metrics, such as accuracy, can provide a partial picture of model performance, especially when there are trade-offs between different aspects of outcome evaluation. The multi-parameter metric can incorporate different performance measures to provide more comprehensive results, the assessment of which can support better prediction.

However, it is important to note that statistical algorithms and models are not universally applicable to all datasets or medical conditions. Different types of data and different clinical questions may require customized approaches. Researchers are, therefore, advised to use a range of models during the training and validation process. This diverse approach allows them to identify those models that show the most promising initial results and the greatest potential for refinement and further development [21]. This work also underscores a well-known tenet in Data Science: the performance of a model depends heavily on the size and quality of the dataset [22].

From a practical standpoint, the work that has been completed can be regarded as a successful proof of concept. In order to derive tangible benefits from DS models, they must be deployed in a real-world setting. According to the established production standards, deploying the model in shadow mode is necessary. In shadow mode, doctors are expected to make independent decisions while the algorithm runs in the background, and its results are compared against the ground truth data [23]. Furthermore, once deployed, AI models must be continuously monitored, and medical professionals must calibrate them to align, among others, intrinsic metrics, such as sensitivity and specificity, with extrinsic metrics, such as patient health outcomes, recognizing that AI development is an iterative process requiring ongoing refinement.

To evaluate an AI solution beyond its predictive value, we need to think about a framework that can be systematically applied across the broad range of AI solutions emerging in healthcare. We need a framework that provides criteria to evaluate the utility, feasibility, and overall clinical impact of an AI solution [24].

During the course of our research, we developed a novel and simple tool to assist clinicians in identifying patients with AML who might benefit from thromboprophylaxis. However, several further steps would lead to widespread score usage in everyday clinical practice. First of all, the score must be externally validated. After that, a prospective study of thromboprophylaxis in patients classified as high risk according to our score should be conducted, especially to learn about the frequency of bleeding complications. In the end, education and clinician training should be conducted.

## 5. Conclusions

In conclusion, the strategic application of Data Science methodologies in medical research and practice has the potential to revolutionize the field, providing more accurate, timely, and personalized insights into patient care. However, careful consideration must be given to model selection, validation, and the proper communication of results to ensure these advancements translate into real-world improvements in healthcare delivery.

## Figures and Tables

**Table 1:** The performance of algorithms on the test set.

Algorithms/Metrics	MLP	Ridge Regression	Naive Bayes	Logistic Regression
AUC	0.749	0.734	0.716	0.697
Accuracy	0.580	0.660	0.596	0.644
Sensitivity	0.773	0.636	0.733	0.682
Specificity	0.554	0.663	0.572	0.639
F1	0.301	0.304	0.309	0.309

## Data Availability

The datasets used and analyzed during the current study are available from the corresponding author on reasonable request.
